# Marketing trials, marketing tricks — how to spot them and how to stop them

**DOI:** 10.1186/s13063-017-1827-5

**Published:** 2017-03-08

**Authors:** Alastair Matheson

**Affiliations:** Toronto, Canada

**Keywords:** Pharmaceutical industry, Marketing, Seeding trial, Marketing trial, Bias, Authorship, Transparency, Disclosure, Research integrity, Guidelines, Checklist

## Abstract

**Background:**

Last this year in this journal, Barbour and colleagues reported a study of “marketing trials” in leading medical journals (*Trials* 2016;17:31). In this commentary I discuss their research, describe new analyses of the study cohort and consider measures to address marketing within academic medical literature.

**Discussion:**

Barbour et al. sought to identify a subgroup of “marketing trials” within leading medical journals, but in reality, nearly all industry-financed trials serve marketing functions, and many exhibit marketing-related features, including biases, in their framing, methodology or reporting. I conducted new analyses of the cohort of Barbour et al., showing that most trials funded exclusively by drug manufacturers had direct involvement of the manufacturer in design, analysis and reporting, and features supportive of product seeding. However, these commercial enterprises were without exception presented to journal readers as academic-led projects, using attributional spin, which should itself be considered an important form of marketing bias. Barbour et al. correctly conclude that commercial bias in industry clinical trials articles often requires expertise to recognize, and in many cases cannot be identified from the published journal report. Several potential remedies are discussed, including independent clinical research, data sharing, improved reporting guidance, improved tools for assessing research quality, reforms to article attribution, submission checklists and new editorial standards.

**Conclusion:**

Medicine’s journals have a responsibility to uphold rigorous scientific and reporting standards, require ready trials data access and ensure the commercial dimensions of research are brought prominently to their readers’ attention. Failure to meet these responsibilities constitutes an enduring threat to the integrity of biomedical literature.

**Electronic supplementary material:**

The online version of this article (doi:10.1186/s13063-017-1827-5) contains supplementary material, which is available to authorized users.

## Background

Last year in this journal, Barbour and colleagues published an analysis of 194 journal articles with the striking claim that “21% of drug trials … in the leading general medical journals had characteristics consistent with the aim of marketing the product” [1]. Their study raises important questions about how marketing works in industry-financed clinical trials and the journal articles which report them, and what measures should be taken to address its influence.

## Discussion

Marketing can be defined straightforwardly as the promotion of any product, service or organization to its customers, but the term “marketing trial” is ambiguous. Its usage is similar to the term “seeding trial”, in which prospective customers are involved in fatuous research to familiarize them with a commercial product, in the hope they will continue to prescribe it after the trial has ended [2–6]. But are Barbour et al. claiming that one fifth of the clinical trials in leading journals, and substantially more in the *Lancet* and *New England Journal of Medicine*, are such cynical enterprises?

The answer is no. Fatuous seeding trials are more likely to be published in lower tier journals, and may not involve controlled designs. Some trials in leading journals have been designed in consultation with regulators to win marketing approval, and while they may be conservative, most are not fatuous. Thus, if the figure of 21% from Barbour et al. were taken as a measure of the prevalence of wholly worthless research in leading journals, it would be an overestimate. Yet conversely, it would be a significant *underestimate* of the prevalence of marketing — and probably marketing bias — within this literature.

### Marketing functions and marketing-related features

To understand the interaction between marketing and clinical trials, it is necessary to shift perspective from “marketing trials”, which define only one point along a spectrum of commercial influence, and map the problem more systematically. In particular, it is helpful to separate the marketing *functions* industry trials perform from their marketing-related *features*, that is, the details of framing, design and reporting which have been commercially configured the better to serve marketing goals.

As Table 1 illustrates, various strategic considerations determine the choice of trials manufacturers undertake. Once the selection is made, however, trials serve numerous marketing functions. Most obviously, they generate reservoirs of data, as a basis for evidence-based medicine, and equally, evidence-based marketing. This is the case for off-label as well as on-label research, and a seemingly innocuous trial exploring a new use of a drug may constitute off-label marketing — indeed, this is sometimes a specific strategy [7].

**Table 1:** Interactions between marketing and clinical trials

General influences on the choice and design of industry trials
• Clinical/commercial product profile, competitor landscape, opportunities and risks • Regulatory requirements • Cost, time, patient recruitment and logistics
Marketing functions of industry trials
• Generating commercially useful data • Engaging, organizing and retaining key opinion leaders • Building relationships with investigators and their institutions, including internationally • Seeding — familiarizing clinicians with use of a product such that they continue using it after the trial • Generating publicity, interest and prestige for a drug and its manufacturer, for instance, through publications, congresses and material for sales representatives • Internal marketing — building company enthusiasm and product understanding
Marketing-related features of industry trials A. Research question
• May be meaningful or fatuous; ambitious or conservative; balanced or loaded; appropriately or inappropriately framed
B. Commercial choices and biases in design, conduct and analysis
• Not all commercial trials are biased, and marketing can be based on unbiased data and reporting • Nonetheless, many industry trials involve commercially expedient methodological choices and biases. These may be unconscious or planned • Randomized studies — multifarious opportunities for commercially expedient decisions and biases, often closely related to the clinical particularities of the trial
C. Commercial choices and biases in reporting
• Non-reporting of unhelpful trials and data • Delayed, obscure or underreporting: choice of journal, website or congress proceedings • Published articles — selective reporting of favourable vs. unfavourable results; inferring greater clinical relevance than the data justify; framing, interpretation, visual spin, rhetoric and conclusions • Overreporting of favourable findings in secondary publications • Attributional spin, highlighting the role of academics and understating that of manufacturers, is endemic in medical journal articles

Trials are also a tool for recruiting and leveraging key opinion leaders (KOLs) [8, 9]. A large trial keeps in place a substantial group of KOLs, maintains high-level interest and helps the company forge relationships with and among KOLs, creating a network of “influencers” to serve the company’s interests. Large long-term trials are also used directly to promote drugs: for example, most angiotensin ﻿receptor blockers had long-term outcomes trials [10–15] which addressed clinically relevant issues but were also used during their courses to generate interest and product differentiation, for instance, at congresses or through journal publications. Finally, trials can be used for product seeding, by spreading the study population thinly across numerous investigator sites, each of which enrolls relatively few patients. Such “seeding” features do not only function, however, to familiarize prescribers with the product. They promote regular contact between the company’s representatives and prescribers, building familiarity and trust and assisting sales not only of the study drug, but of other products. They also facilitate “internal marketing”, that is, generating enthusiasm for the product within the company; they help local teams acquire greater knowledge of the product; and when recruitment is a challenge, they may enable trials to be completed more quickly. There may also be regulatory considerations; for instance, some countries require their own nationals to be involved in pivotal trials for a license to be granted, and the need for an ethnically diverse population may also increase the number of study sites and countries [16].

None of these marketing functions, with the exception of seeding, necessarily compromises the quality of the science in industry trials. The best industry trials of promising new drugs, including many pivotal regulatory trials where the company stands to lose billions if the study fails, have robust designs and are objectively reported. In other cases, however, marketing encroaches into the trial’s features, influencing the framing of research questions, steering the design choices, driving the accumulation of biases and shaping the reporting of results (Table 1) [17–25]. The foremost difficulty with industry research is not the fatuous marketing trial, but the coexistence within the same clinical studies of defensible science and subtle marketing spin, rendering the results harder to evaluate, but easier to sell.

### Corporate authors, widespread seeding and attributional spin

Who is responsible for commercial encroachment into this science? To some degree, the academic authors are culpable, whether through lack of awareness or poor judgement. Many such individuals are trusted partners who enjoy financial relationships with the manufacturers [26, 27] and know that if they disappoint their patrons, they may not be invited back. The primary culprits, however, are the companies themselves, who in most cases are proprietors and corporate authors of this research, and its one essential component: the academic authors and institutions recruited into these projects are replaceable by others [28]. To refer to companies as mere “sponsors” of these trials or providers of “funding” or “support” conceals their true role, and such terms should be discarded.

To demonstrate the extent of corporate involvement, it is first necessary to distinguish between true industry projects funded and instigated by manufacturers, and studies that merely received a degree of industry funding. Barbour et al. kindly shared their database to enable me to undertake this analysis (see Additional file 1). A total of 68 trials in their cohort of 194 studies received exclusive manufacturer funding, and in the large majority of these trials, the company was involved directly in design (82%), analysis (83%) and also reporting (76%), deploying company employees as coauthors (88%) and using trade writers (69%). Lundh and colleagues have previously reported similar findings in this journal for a cohort of articles published in the *Lancet* [29]. Furthermore, designs suggestive of seeding were not limited to a few “seeding trials” but were commonplace among the manufacturer-funded trials in the cohort. The median number of investigator centres in trials funded exclusively by the drug manufacturer was 111, but remarkably, the median number of patients randomized per centre was only 9. These findings suggest that seeding is a widespread feature of manufacturer-financed clinical research.

Yet while these trials are corporate projects, they are not presented to readers as such. The most visible form of bias in this literature is *attributional bias*, wherein the role of the academic participants in the study is highlighted and industry’s downplayed [28, 30, 31]. This ensures industry work is presented to readers under the lead authorship of credible academics, and by reducing the impression of commercial influence, may also increase the prospect of journal publication [32]. Barbour et al. did not investigate attributional spin, but I identified all the industry-financed articles in their cohort with both academics and industry employees as coauthors, which numbered 70 in total (see Additional file 1). Among these, all 70 had academic lead authors: there was not a single article where an employee of the company fronted the work. This is the first time the systematic fronting of industry projects by academics has been reported for a defined cohort of articles.

### Spotting marketing and bias

Readers of journal articles that report clinical trials should always check for industry funding, industry involvement in research and reporting, ties between academic authors and the manufacturer and academic lead authorship. When present, these features should alert readers to the possibility that marketing is at work, and mandate careful scrutiny of the article. But what of the details of framing, methodology and reporting? Can readers be provided with a simple checklist to spot bias at this level?

The answer, unfortunately, is no. As Barbour et al. correctly conclude, “individual trials have a unique combination of features reported in the journal publications. The pattern of features makes marketing-influenced studies difficult to identify by the average reader.” Certain features, such as uninformative comparisons, noninferiority designs, surrogate or composite endpoints and speculative conclusions, should raise concerns, but none points with certainty to commercial bias, and in many cases they are reasonable or agreed with regulators. Positive spin in the conclusions affects noncommercial as well as commercial research, and is in any case encouraged by journals [33, 34]. Expert reading of articles by clinicians in the field and authorities in trials design can identify many flaws, and readers should seek independent commentaries and online forums for further insight. However, perhaps the greatest difficulty with commercial bias is that it can prove undetectable even by experts on the basis of the information reported in the article, and may only become fully apparent on rare occasions when the manufacturer’s study reports and database trial data are independently scrutinized [35]. Ultimately therefore, the question of how to spot marketing tricks has a troubling answer. In many cases, they *cannot be spotted*, and certainly not by the everyday prescribers who are this literature’s primary targets.

### Solutions

Barbour et al. rightly argue that only independent research can fully remedy these problems, but in its absence many actions are possible. The concept of research integrity should be recalibrated such that commercial secrecy, spin and bias are viewed with the same gravity as individual falsification and fraud. There have recently been encouraging steps towards data sharing [36–38], but far more must be done to ensure academics have ready access to all commercial trials databases, study reports and protocols, including data from past trials for licensed products, and without undue red tape.

Among the various other possible interventions, I briefly consider two. Firstly, two important resources, the CONSORT trial reporting standards and the Cochrane Risk of Bias Tool [39, 40], require further development. Good CONSORT compliance does not necessarily point to high trial quality [41], and both CONSORT and the commonly used trial assessment and bias tools have been criticized for lack of scope and detail [42–44]. Commercial biases may escape detection not only because they are subtle or hidden in the published article, but because they are poorly captured by the available bias categories. This is particularly the case with what might be termed “design bias” or “hard-wired bias” involving details such as the choice of comparator, drug doses, dose escalation schedules, efficacy criteria and adverse event coding [21, 44, 45]. The *Cochrane Handbook for Systematic Reviews* addresses this difficulty in a number of ways and the Risk of Bias Tool includes an “other biases” category [44], but the issue remains poorly articulated. In some cases, CONSORT compliance and favourable bias scores may have the unintended effect of conferring credibility on research that does not deserve it, and it is notable that advocates for the commercial writing trade draw attention to the CONSORT compliance of the articles they develop as evidence of their validity [46, 47]. These considerations challenge the trials community to improve the guidance for reporting trials and assessing their quality, particularly in the setting of industry research. The sheer diversity of potential biases in commercial trials and their close relationship with the clinical particularities of the trial setting may make this a thorny task.

Secondly, what of Barbour’s primary concern, the journal articles themselves? Many journals and their publishers have substantial conflicts of interest in respect of industry, whose trials fill their pages and whose reprint purchases swell their revenues [48–50]. Journals dependent on industry patronage have a financial incentive to publish commercial research even when its importance is low, and to allow trial reports to be framed and communicated in a fashion which assists their marketing functions. Yet journals are the custodians of the academic medical canon and have both responsibility and power to demand open data, rigorous scientific and reporting standards and appropriate attribution. Not all journals are fulfilling these responsibilities with adequate scientific zeal, and because this problem is enduring, systematic and potentially dangerous to patients, it should be considered as important a challenge to the integrity of academic medical literature as research misconduct.

There have been calls for journals to cease publishing industry trials [51, 52], but a more realistic goal is to raise journal standards. The International Committee of Medical Journal Editors (ICMJE) guidelines recommend a number of steps which improve the transparency of commercial literature, but they remain permissive of pharmaceutical marketing [53]. No article should be deemed acceptable for journal publication without the accompanying publication of the trial protocol, completed to SPIRIT standards [54], an unsparing account of the work’s scientific and clinical limitations, and prompt access to patient-level data and clinical study reports. Furthermore, since the flaws and biases in this literature are often difficult for readers to identify, a stronger onus should be placed on the trials’ own stakeholders to report any features which might favour the product, as a matter of research integrity. Altman and Moher have called for journals to require lead authors to make a signed declaration of the work’s honesty, accuracy and transparency [55], and building upon this approach, journals should require authors and product manufacturers to supply detailed information on the commercial provenance and design features of the work, a task that could be accomplished using a checklist (Table 2).

**Table 2:** Developing an author-completed checklist for journal articles reporting industry trials

Items for inclusion in checklist
A. Commercial aspects of the study • Identification of all stakeholders, including manufacturer, agencies, academic institutions, authors, contributors • Who instigated the research? • How was it financed? Total, majority or minority finance from manufacturer? • Identify main product of commercial interest • Frank statement of the commercial relevance of the research (e.g. securing approval, demonstrating advantage over competitor, providing practical experience of product) • Details of intellectual property/data ownership and summary of data sharing plan • Details of all recent payments by manufacturer to participating institutions, agencies, authors and contributors • Confirmation that all author/contributor interests are registered on a public website • Public URL for all commercial publications plans in which the article is listed • Consent for journal to publish number of reprint/eprint sales for article B. Study design, analysis, interpretation and reporting • Clear characterization of company and agency role • Confirmation of compliance with reporting guidelines (CONSORT guidelines should be upgraded) • Provide point-by-point response to checklist of design features, identifying those potentially favouring the product or limiting the applicability of the study. Examples: Patient selection; Choice of control treatment; Doses and regimens; Run-in period; Assessment criteria; Safety definitions; Cut-off points for data inclusion; Deviations from protocol; Early stopping; etc.
Responsibilities for journals
• Ensure full checklists are provided with submission, signed off by the corresponding author and company representative • Ensure peer reviewers are provided with completed checklist • Use checklist to: ° Ensure potential methodological biases and design features likely to favour the product or limit the study’s applicability are fully described and prominently tabulated in the published article ° Check commercial roles and goals are prominently described in the article ° Check attribution, labelling and disclosures reflect true role of the company, e.g. name manufacturer in title of article; place article in designated “industry trials” section of journal • Publish completed checklists online for published articles • Publish reprint purchase numbers for all articles • Consider corrections, retraction, referral to research integrity office and legal action if checklist proves to have been inaccurately completed

The uncomfortable truth, however, is that journals and publishing corporations inured to industry patronage are unlikely to seek reform of a culture in which they prosper. One final possibility might therefore be the development of a new editorial standard, supplementary to that of the ICMJE and more exacting, for journals committed to rigorous science and reporting. If readers came to recognize the standard and its logo as a mark of scientific stringency, this might stimulate wider uptake by journals and raise the quality of clinical trial reporting.

## Conclusions

Pharmaceutical research produces innovative, life-saving medicines which should be celebrated, but it is also undermined by marketing, leading to trials of marginal scientific interest, widespread product seeding and the systematic penetration of marketing bias, much of it subtle or hidden, into the broader clinical trials literature. Manufacturers are de facto corporate authors of much of this research, but the published literature is spun to give the impression of academic-led projects. The “marketing” or “seeding” trial represents only one, variably defined point within a spectrum of undesirable commercial influence. Barbour et al. rightly argue that the most effective solution to these challenges is independent research, but in addition, ready data access, upgraded CONSORT guidance, more detailed bias assessment tools and more exacting editorial requirements have a role to play. Journals have a critical responsibility to enforce exacting scientific and communicative standards, but many titles which prosper from industry patronage are failing in this duty, to the detriment of medicine.

I congratulate Barbour et al. on their important study. They describe their research as “more suited to start a debate than to settle it”, and my analysis and the remedies I have considered follow in the same spirit. I encourage others to join the conversation.

## Additional file

Additional file 1: Appendix: new a﻿nalyses of the study cohort of Barbour et al. (ZIP 214 kb)

## Abbreviations

CONSORT: Consolidated Standards of Reporting Trials
ICMJE: International Committee of Medical Journal Editors
KOL: Key opinion leader
SPIRIT: Standard Protocol Items: Recommendations for Interventional Trials
