# Supplementary material for: Marketing trials, marketing tricks — how to spot them and how to stop them
Source: Trials. 2017 Mar 8;18:105. doi: 10.1186/s13063-017-1827-5 (PMC5341186; doi:10.1186/s13063-017-1827-5)
Supplement: Additional file 1: — Appendix: new a﻿nalyses of the study cohort of Barbour et al. (ZIP 214 kb) [file 13063_2017_1827_MOESM1_ESM.zip › 13063_2017_1827_MOESM1_ESM/Matheson Trials Appendix revisedR2.docx]

**Appendix**

**Introduction**

In this Appendix I describe new analyses I performed on Barbour et al’s cohort of 194 clinical trial articles from 6 leading medical journals.[1]

In Barbour et al’s original analysis, 6 experts assessed each article in the cohort and identified those they believed were likely marketing trials. When 4 experts or more concurred, the article was classified as a “suspected marketing trial”. The experts made their determinations subjectively, but on the basis of 6 motifs. These were: manufacturer involvement in design, analysis and reporting; seeding features; misleading abstracts; and finally conclusions focusing on surrogate and composite endpoints. Separately, a blinded group of investigators recorded numerous features of all the articles in the cohort. Finally, the suspected marketing trials were screened against this database to characterize their features. Readers are referred to the original publication for full details of the methodology.

Barbour et al kindly shared their data with me to enable me to investigate two features of industry trials not assessed in their analysis. The first of these was exclusive funding by the manufacturer. Barbour et al recorded whether or not studies had received industry funding, but did not isolate studies in which the manufacturer was the sole funding source credited. This distinction is important because studies funded exclusively by manufacturers are likely to be true commercial projects, owned and instigated by companies themselves, whereas those with additional funding sources may have a noncommercial provenance and serve a range of interests.

The second parameter I investigated was attributional spin – that is, attribution which directs the reader’s attention to the role of academics in these projects, as opposed to the companies themselves. To assess attributional spin, I assessed how many industry-funded articles placed an academic at the front of the author byline.

**Methods**

I examined all the articles in Barbour et al’s cohort, and classified them according to whether they were exclusively, partly or unfunded by the drug manufacturer. When this information was not provided in the article, I obtained it from the trial’s online registration. I tabulated my findings in an Excel spreadsheet and compared exclusively-funded, part-funded and non-industry trials, according to the parameters already recorded in Barbour et al’s database. For statistical comparisons, I used Fisher’s exact test or Mann-Whitney U-tests, conducted using online resources.[2,3]

To assess attributional spin, I inspected the author bylines of every article in the cohort, and identified the subset of articles which had both academics and company employees as authors. I then counted the number of articles within this group in which an industry employee was listed as the first author on the byline.

**Results**

Of 106 studies classified by Barbour et al as industry funded, 68 exclusively credited the drug manufacturer with funding. Of the 41 studies classified by Barbour et al as likely marketing trials, 37 (90%) were exclusively funded by the manufacturer.

I examined the authorial role of companies in these 68 trials (Table 1). Compared with part-industry funded and unfunded trials, I found that exclusively-funded trials exhibited very high levels of manufacturer involvement in design and analysis (82% and 83% of trials, respectively), the use of company employees as coauthors (76%) and the use of writers to develop the manuscript (69%) (Figure 1). Organizational features likely to be supportive of product seeding were rare in non-industry studies, but were apparent in both partially-funded and, especially, sole-funded industry trials (Figure 2). The median number of centres used in trials exclusively funded by the drug manufacturer was 111, but the median number of patients randomized per center in these trials was only 9.

Since 37 of the 41 “YES” trials identified by Barbour et al were exclusively funded by manufacturers, I also assessed whether the characteristics of “YES” trials might simply have been features of exclusively-funded industry studies per se. As shown in Table 2, I found that there was no statistically significant difference between the “YES” trials and “NO” trials with exclusive manufacturer funding, in respect of the manufacturer’s involvement in design, analysis and reporting. Seeding features were present in all industry trials, but were most pronounced in “YES” trials. The other features of “YES” trials identified by Barbour et al remained unaffected by this analysis.

With respect to attributional spin, I found a total of 70 articles (60/68 exclusively-funded and 10/38 partly-funded articles) with both academic and industry employee-coauthors. Of these 70 articles, the number with an industry employee listed as the first author was zero.

**Discussion**

This new analysis of Barbour et al’s database found that exclusive manufacturer funding was strongly associated with direct manufacturer involvement in all stages of research, analysis and reporting. Similar findings were previously related for a cohort of *Lancet* articles by Lundh and colleagues.[4] Furthermore, organizational features likely to be supportive of product seeding were not restricted to a subset of “marketing trials”, but were a general feature of manufacturer-financed clinical research.

The analysis also found evidence of attributional spin. In the entire cohort, there was not a single industry-funded article with both industry and academic coauthors in which an industry employee was the lead author. The use of academic authors to front industry projects had previously been noted,[5] but this is the first time this observation has been documented in a defined cohort of articles. With a larger cohort, some degree of employee-lead authorship would be expected, especially in the settings of small company, biotechnology or proof of concept research, but this practice is likely to be unusual for clinical trials from phase II onward, when these are funded by mid- to larger pharmaceutical corporations.

Considering these new analyses of Barbour et al's database together, a culture is revealed in which manufacturers have direct authorial involvement as corporate entities at every stage of the conception, design, analysis and reporting of their solely-owned projects; but in which the attribution of these projects is spun to downplay the manufacturers’ role and highlight academic recruits. Their names bolster the credibility of this research with journal readers.[6,7]

There are several limitations to this analysis. It was not prospective, and I undertook it with an expectation of the likely outcome from my knowledge of marketing practices. Furthermore, I categorized and counted the articles single-handedly. For these reasons, these findings should be confirmed in new, prospective research by academic groups. The findings were so unambiguous, however, that they would not be substantively affected by minor counting errors.

I also assessed the validity of Barbour et al’s findings regarding the features of marketing trials identified in their study. My results suggested that the authorial role of companies in this research is not, as suggested by Barbour et al, a feature specific to “suspected marketing trials” as designated in their analysis, but is in fact a general feature of research funded exclusively by manufacturers. Features supportive of seeding also appear to be a general property of manufacturer-run research, although they were more pronounced in suspected marketing trials.

I thank Barbour et al for sharing their data and enabling me to make these additional analyses.

**References**

1. Barbour V, Burch D, Godlee F, Heneghan C, Lehman R, Perera R, et al. Characterisation of trials where marketing purposes have been influential in study design: a descriptive study. Trials. 2016;17:31.

2. GraphPad Software, Inc. QuickCalcs. <http://www.graphpad.com/quickcalcs/> Accessed 6 December 2016.

3. University of Saarland. Wilcoxon-Mann-Whitney Test Calculator. <https://ccb-compute2.cs.uni-saarland.de/wtest/?id=www/www-ccb/html/wtest>. Accessed 6 December

2016.

4. Lundh A, Krogsbøll LT, Gøtzsche PC. Sponsors' participation in conduct and reporting of industry trials: a descriptive study. Trials. 2012;13:146.

5. Ross JS, Hill KP, Egilman DS, Krumholz HM. Guest authorship and ghostwriting in publications related to rofecoxib: a case study of industry documents from rofecoxib litigation. JAMA. 2008;299:1800-12.

6. Matheson A. Ghostwriting: the importance of definition and its place in contemporary drug marketing. BMJ. 2016;354: i4578

7. Matheson A. The disposable author: how pharmaceutical marketing is embraced within medicine’s scholarly literature. Hastings Center Report. 2016; doi10.1002/hast.576.

**Figures and tables**

Appendix Figure 1. Company involvement in trials design, analysis and reporting in exclusively manufacturer-funded, partly manufacturer-funded and non-industry trial publications.

Appendix Figure 2. Number of countries and seeding features in solely manufacturer-funded, partly manufacturer-funded and non-industry trial publications.

Table 1. Comparison of exclusively manufacturer-funded, partly manufacturer-funded and non- manufacturer-funded trials.

|  | All trials (n=194) | Sole-industry funded trials (n=68) | Part-industry funded trials (n=38) | No industry funding (n=88) | *p* value (sole vs. part funding) | p value (sole vs no funding) | P value (part vs no funding) |
| --- | --- | --- | --- | --- | --- | --- | --- |
| Manufacturer involved in the design of the study^ | 71 (37) | 56 (82) | 10 (26) | 5 (6) | <0.0001 | <0.0001 | 0.0021 |
| Manufacturer involved in the data analysis^ | 66 (34) | 57 (83) | 7 (18) | 2 (2) | <0.0001 | <0.0001 | 0.0032 |
| Manufacturer involved in the reporting  of the study^ | 64 (33) | 52 (76) | 8 (21) | 4 (5) | <0.0001 | <0.0001 | 0.009 |
| Medical writer used^ | 75 (39) | 47 (69) | 10 (26) | 18 (20) | <0.0001 | <0.0001 | 0.4897 |
| Byline author from the product manufacturer^ | 76 (39) | 60 (88) | 10 (26) | 6 (7) | <0.0001 | <0.0001 | 0.0064 |
| Company employee as lead author^ | 1 | 0 | 0 | 1 | - | - | - |
| Median number of patients screened* | 1103 | 1210 | 1021 | 1134 | 0.5572 | 0.6826 | 0.7305 |
| Median number of patients randomized* | 620 | 771 | 645 | 371 | 0.2941 | 0.0037 | 0.215 |
| Median number of countries* | 1 | 13 | 2 | 1 | <0.0001 | <0.0001 | 0.0003 |
| Median number of centres* | 28 | 111 | 43 | 9 | 0.0001 | <0.0001 | <0.0001 |
| Median number of patients screened per centre* | 57 | 12 | 55 | 171 | <0.0001 | <0.0001 | 0.0047 |
| Median number of patients randomized per centre* | 19 | 9 | 19 | 58 | <0.0001 | <0.0001 | 0.0053 |

^ Percentages in brackets. Statistical comparisons by Fisher’s exact test.

* Statistical comparisons by Mann-Whitney U-test.

Table 2. Comparison of YES trials (i.e. suspected marketing trials) with exclusively manufacturer-funded, partially- manufacturer-funded and non- manufacturer-funded NO trials

|  | YES trials (n=41) | NO, sole funded (n=19) | NO, part funded (n=32) | NO, non-funded (n=88)) | *p* value (YES vs NO sole funded) | *p* value (YES vs. NO part funded) | *p* value (NO sole vs. NO part funded) |
| --- | --- | --- | --- | --- | --- | --- | --- |
| Manufacturer involved in the design of the study^ | 34 (83) | 15 (79) | 7 (22) | 5 (6) | 0.7298 | <0.0001 | <0.0001 |
| Manufacturer involved in the data analysis^ | 35 (85) | 14 (74) | 5 (16) | 2 (1) | 0.3007 | <0.0001 | <0.0001 |
| Manufacturer involved in the reporting  of the study^ | 33 (80) | 12 (63) | 5 (16) | 4 (2) | 0.2021 | <0.0001 | 0.0008 |
| Speculation or generalized phrasing in conclusions^ | 24 (59) | 7 (37) | 11 (34) | 33 (38) | 0.1664 | 0.0059 | 1.0000 |
| Median number of patients screened* | 1338 | 833 | 1021 | 1134 | 0.3656 | 0.2543 | 0.5996 |
| Median number of patients randomized* | 1195 | 636 | 435 | 371 | 0.0649 | 0.0122 | 0.6503 |
| Median number of countries* | 19.5 | 11 | 1 | 1 | 0.0213 | <0.0001 | 0.0231 |
| Median number of centres* | 171 | 42 | 29 | 9 | 0.0002 | <0.0001 | <0.0001 |
| Median number of patients screened per centre* | 11 | 45 | 55 | 170.5 | 0.0517 | <0.0001 | 0.0049 |
| Median number of patients randomized per centre* | 9 | 17 | 18.5 | 58 | 0.4455 | 0.0002 | 0.0048 |

^ Percentages in brackets. Statistical comparisons by Fisher’s exact test.

* Statistical comparisons by Mann-Whitney U-test.
