# Supplementary figures and images for: Marketing trials, marketing tricks — how to spot them and how to stop them
Source: Trials. 2017 Mar 8;18:105. doi: 10.1186/s13063-017-1827-5 (PMC5341186; doi:10.1186/s13063-017-1827-5)

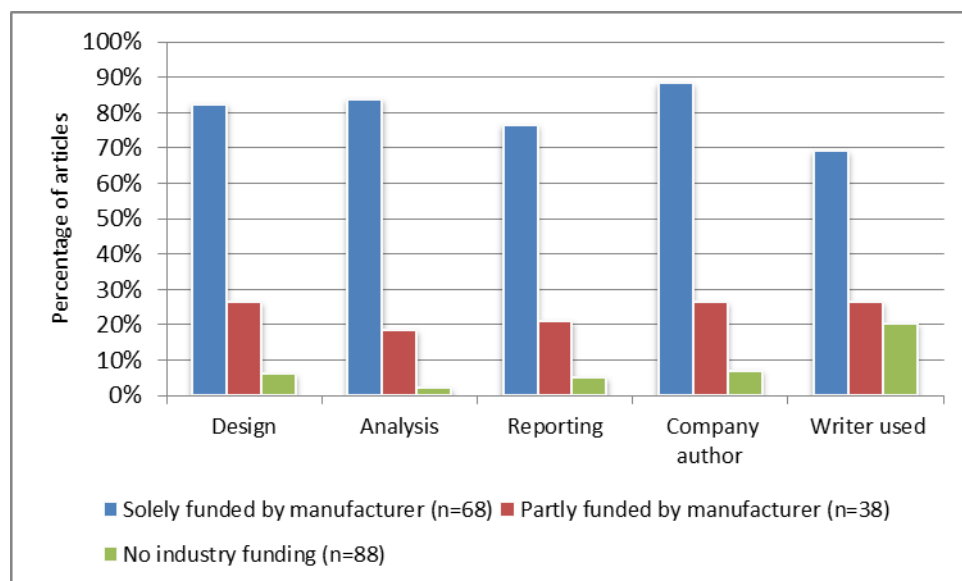

Supplement: Additional file 1: — Appendix: new a﻿nalyses of the study cohort of Barbour et al. (ZIP 214 kb) [file 13063_2017_1827_MOESM1_ESM.zip › 13063_2017_1827_MOESM1_ESM/Matheson Trials Appendix Figure 1R2.pdf]

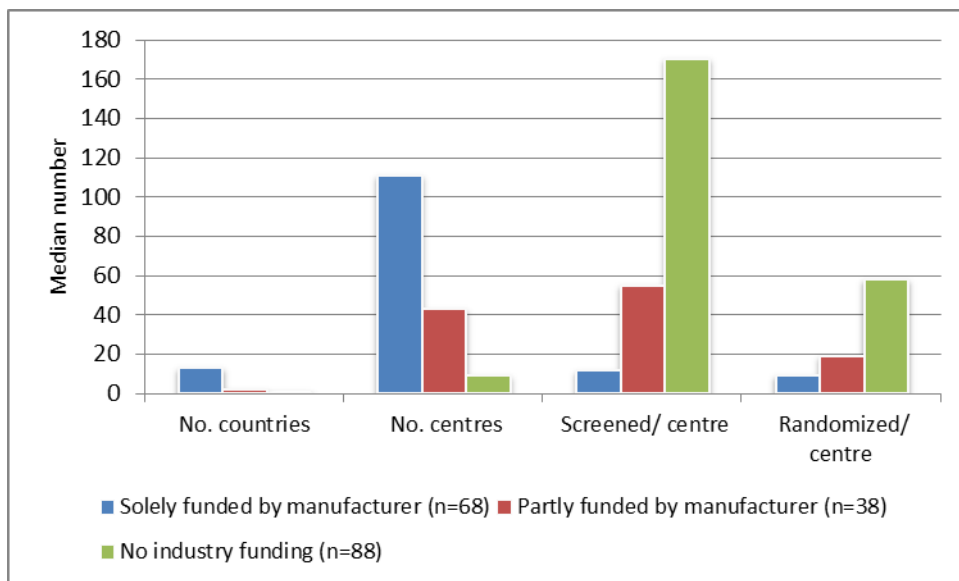

Supplement: Additional file 1: — Appendix: new a﻿nalyses of the study cohort of Barbour et al. (ZIP 214 kb) [file 13063_2017_1827_MOESM1_ESM.zip › 13063_2017_1827_MOESM1_ESM/Matheson Trials Appendix Figure 2R2.pdf]
